# Parameter Adaptive Network for Large-Scale Neural In-Loop Filtering in Versatile Video Coding

**DOI:** 10.3390/s26092808

**Published:** 2026-04-30

**Authors:** Yuansheng Wu, Fan Cai, Xiaodan Song, Xuguang Zuo

**Affiliations:** 1 National Key Laboratory of Complex Aviation System Simulation, Southwest China Institute of Electronic Technology, Chengdu 610036, China; wysefan@163.com; 2 Guangzhou Institute of Technology, Xidian University, Guangzhou 510555, China; fancai@stu.xidian.edu.cn; 3 NetInt, Shanghai 201203, China; zuoxuguang0519@163.com

**Keywords:** versatile video coding, in-loop filtering, adaptive parameter, gradient, convolution neural network

## Abstract

Efficient in-loop filtering is critical for the latest video-coding standard, versatile video coding (H.266/VVC). While parameter-adaptive mechanisms are effective in traditional adaptive loop filters and overfitted small-scale neural in-loop filtering, there is difficulty when deploying them in large-scale models since both schemes require explicit parameter signaling within the bitstream, leading to prohibitive overhead. Existing parameter-generation networks avoid these transmission costs but introduce an excessive number of parameters. To address this, we propose modeling convolutional parameters as a linear combination of pre-trained kernels, where weights are adaptively estimated via input-driven attention. Specifically, we propose a multi-scale parameter-adaptive convolution and its extension, with side information, enabling parameter adaptation without transmission overhead or significant computational costs. Furthermore, we have designed an efficient parameter adaptive in-loop filtering network with the proposed convolutions to balance parameter efficiency and reconstruction performance. To improve the distortion guidance provided by side information, we have incorporated gradient information. Experiments on VTM-11.0 demonstrate {7.89%, 18.25%, and 19.15%} bitrate savings for {Y, U, and V} components, outperforming fixed-parameter baselines by an average of 1.41% with negligible computational overhead.

## 1. Introduction

**Figure 1:**
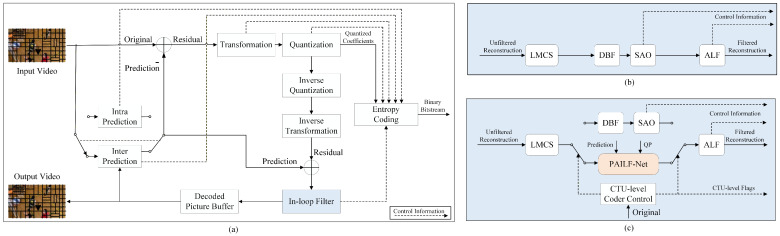
Overview of the H.266/VVC encoding framework and the integration of the proposed in-loop filtering process. (**a**) The VVC encoding pipeline. (**b**) The standard VVC in-loop filtering module. (**c**) The modified in-loop filtering framework incorporating the proposed PAILF-Net.

Over the past few decades, video and image technologies [1,2,3,4] have experienced explosive growth. In particular, the proliferation of surveillance systems and their diverse applications have imposed a significant burden on transmission bandwidth and data compression algorithms [5,6,7,8,9]. This demand for higher efficiency has driven significant advancements in video-coding standards. H.266/VVC [10], the latest standard developed by the joint video experts team (JVET) of ITU-T and ISO/IEC, achieves remarkable compression efficiency, providing a bitrate reduction of over 50% compared to its predecessor, H.265/HEVC [11]. Similar to previous standards, VVC employs a block-based hybrid prediction and transforms coding frameworks to exploit spatial and temporal redundancies, as shown in Figure 1a. However, the lossy nature of this framework inevitably introduces blurring, blocking, and ringing artifacts in reconstructed frames prior to filtering, which significantly degrades both subjective and objective quality metrics.

To restore artifacts and enhance the reconstructed image quality, VVC employs several in-loop filtering (ILF) approaches, including luma mapping with chroma scaling (LMCS) [12], the deblocking filter (DBF) [13], sample adaptive offset (SAO) [14], and the adaptive loop filter (ALF) [15]. These filters are applied sequentially, as illustrated in Figure 1b. Primarily based on traditional signal processing techniques, these hand-crafted filters improve both reconstructed visual quality and objective performance metrics. Notably, the ALF exhibits superior performance compared to the others, primarily due to its inherent parameter adaptation capability.

In recent years, learning-based in-loop filtering has achieved significant performance gains. The initial work employed shallow residual CNNs, e.g., VRCNN [16], which were subsequently succeeded by deeper and more sophisticated architectures [17,18,19,20]. Considering the video compression scenario, researchers have introduced various strategies, including the use of auxiliary side information [19,21,22,23,24,25], inter-frame filtering [26], rate-distortion optimization [19,21,27], and multi-frame processing [22]. Despite their promising results, these methods often lead to increased computational costs for generalization. To facilitate deployment on edge devices, efforts have focused on lightweight designs, e.g., low-complexity CNNs [27,28,29] and DILF [23] with dynamic network structures, as well as unified QP-dependent models [25,30,31] for memory efficiency. However, this typically comes at the cost of compression efficiency. Meanwhile, the JVET [32] is investigating the standardization of neural ILF, classifying these approaches [33,34] into high operation point (HOP), low operation point (LOP), and very low operation point (VLOP) categories to meet diverse demands, balancing performance against complexity. In this paper, we classify networks like HOP as large-scale models characterized by a massive number of parameters and high complexity, albeit with superior performance.

Despite their advantages, most of these existing approaches employ fixed parameters after training, which lacks adaptivity. Recent studies have begun to address this limitation. For small-scale networks, MSR [35] utilizes a CNN to generate multiple hypotheses, which are then linearly superimposed by minimizing the mean squared error (MSE). The linear coefficients are explicitly signaled in the bitstream. Similarly, the JVET [32,36] are investigating content-adaptive LOP/VLOP loop filters by overfitting pre-trained models and transmitting parameter residuals, which significantly improves performance while maintaining low inference complexity. However, applying these schemes to large-scale networks remains challenging as they rely on explicit parameter signaling, which incurs prohibitive overhead for large models. Furthermore, these methods often require additional training stages. For large-scale networks, Meta-ILF [37] avoids such signaling overhead by dynamically generating weights; however, it introduces a complex auxiliary network that requires a significant number of additional parameters and is difficult to train effectively.

In this paper, we propose a parameter adaptive in-loop filtering network (PAILF-Net), which enables parameter adaptation without additional signaling overhead. Importantly, it achieves negligible computational cost with a moderate increase in the parameters. The core idea is to improve classical convolutions with a multi-scale attention mechanism to adaptively aggregate multiple parallel kernels, and this has been inspired by advancements in dynamic neural networks [38]. Specifically, we have developed a multi-scale parameter-adaptive convolution (MSPAConv) and an extended variant, side-information-modulated MSPAConv (S-MSPAConv), incorporating quantization parameter adaptation and side-information guidance. Furthermore, we have integrated these modules into a conventional CNN-based in-loop filtering baseline to construct PAILF-Net using MSPAConv and S-MSPAConv residual blocks. Additionally, we have introduced gradients as side information to guide the distortion restoration. The main contributions of this paper are as follows:

- We propose two novel convolutional layers, MSPAConv and S-MSPAConv, which facilitate parameter-adaptive in-loop filtering without requiring bitstream signaling, thereby achieving efficiency with negligible computational overhead and moderate parameter growth.
- We have developed the PAILF-Net architecture for in-loop filtering by incorporating strategic design principles to balance low parameter counts and computational complexity. Furthermore, we have leveraged gradient information as side information to enhance distortion guidance.
- We have integrated the proposed PAILF-Net into VVC for performance evaluation, as shown in Figure 1c. Extensive experiments demonstrate that our PAILF-Net effectively improves reconstruction quality while maintaining negligible computational overhead.

## 2. Related Work

### 2.1. Neural In-Loop Filtering

Neural network-based in-loop filtering has advanced significantly to mitigate compression artifacts and enhance coding efficiency. Early pioneering approaches employed shallow residual CNNs, such as VRCNN [16]. Then, subsequent works have paved the way for deeper and more sophisticated architectures, including residual highway CNNs [17] for efficient information flow, dense residual CNNs [39] for multi-level feature extraction, and attention-based squeeze-and-excitation networks [18] and multi-density CNNs [19]. Recent advances, most notably SwinIR [20], have further explored the potential of transformer architectures. These innovations are complemented by the integration of auxiliary side information into compression systems, e.g., residual signals [19,21,22], partition information [18,27], prediction data [23,24], frame types [25], and boundary strength [23]. Furthermore, performance has been improved by optimization strategies such as rate-distortion optimization and multi-frame filtering [21,22].

For practical deployment, various lightweight designs have been proposed to balance performance and complexity, including low-complexity CNNs [28,40], separable convolutions [28], unified QP-dependent models [25,30,31], shallow or deeper CNNs for different bit-rate scenarios [18,27], lookup tables [29], and dynamic filtering [23]. The JVET is actively investigating neural network-based ILF for standardization, categorizing models into HOP, LOP, and VLOP categories based on their computational constraints. These investigations demonstrate that both large-scale and small-scale models address distinct application requirements. From the perspective of adaptivity, however, the model parameters in most approaches remain fixed during inference. Since image contents, textures, and distortion characteristics vary significantly, such fixed paradigms inherently limit the expressive capacity of neural networks.

### 2.2. Parameter Adaptive In-Loop Filtering

Parameter-adaptive ILF techniques have been extensively explored in video coding. Notably, ALF [15] enhances subjective quality by applying a Wiener-based filtering process, in which optimal filter coefficients are computed via a least-squares method at the encoder and explicitly signaled to the decoder. Compared to the static fitlers, e.g., DBF [13] and SAO [14], which rely on predefined rules to mitigate specific artifacts, ALF offers superior restoration by employing a content-adaptive Wiener filter that minimizes the reconstruction error, effectively recovering high-frequency details that simpler filters often overlook.

For small-scale networks, Ding et al. [35] proposes a neural adaptive loop filter based on multi-hypothesis sample refinement, which first generates multiple hypotheses via a CNN and subsequently estimates adaptive weighting coefficients. These coefficients require explicit signaling and bitstream compression. Similarly, the JVET has investigated content-adaptive in-loop filtering for LOP and VLOP [32,36], employing model overfitting at the encoder while maintaining low inference complexity. In these methods, the overfitted parameters are signaled within the bitstream, which necessitates additional per-sequence or per-frame training. However, these approaches are impractical for large-scale models, as the prohibitively large number of parameters imposes significant transmission overheads. Alternatively, Meta-ILF [37] circumvents direct signaling by dynamically generating convolution parameters using an auxiliary network conditioned on the QP to enhance adaptivity without requiring parameter transmission. Despite these benefits, the auxiliary generation network significantly increases the overall model size, potentially introducing inference latency and training instability.

In this paper, drawing inspiration from advancements in dynamic neural networks [38], we model the convolutional parameters as a linear combination of pre-trained expert kernels, wherein the adaptive weights are derived via a lightweight network driven by the input data. Consequently, the proposed method facilitates transmission-free adaptation and enhances reconstruction quality while incurring only negligible computational overhead and moderate increases in parameter count.

## 3. MSPAConv: Multi-Scale Parameter-Adaptive Convolution

In this section, we first elaborate on the architecture of the proposed MSPAConv and its adaptive parameter mechanism. Subsequently, we present the side-information-modulated MSPAConv (S-MSPAConv) and analyze its adaptive QP-modulation properties. Finally, we evaluate and compare the computational complexity and parameter overhead among standard convolutions, MSPAConv, and S-MSPAConv.

### 3.1. MSPAConv Structure

**Figure 2:**
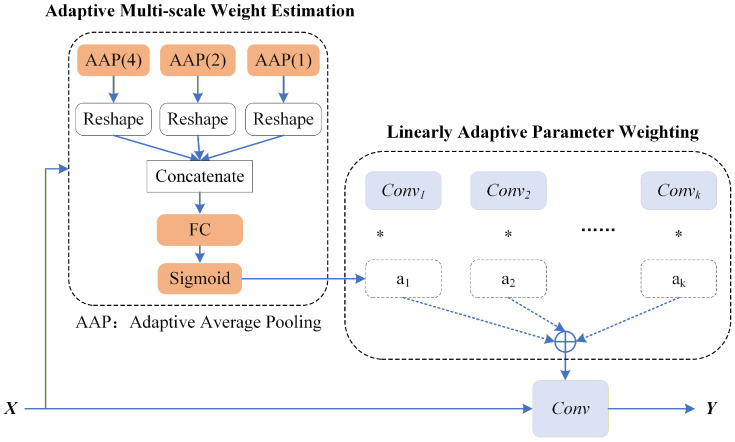
Architecture of the proposed MSPAConv. AAP denotes adaptive average pooling, and FC represents the fully connected layer. $Conv_{i}$ denotes the *i*th convolution kernel, while $a_{i}$ corresponds to the adaptive weight inferred from the multi-scale weight-estimation module. ∗ denotes element-wise multiplication. Since these adaptive weights are derived from input features available at both the encoder and decoder, they do not require additional signaling or compression.

To enable parameter-adaptive ILF while maintaining a lightweight design, we propose MSPAConv, inspired by dynamic convolutional networks. The core concept involves generating convolutional parameters as a linear combination of multiple parallel kernels. These weights are adaptively derived from the input feature maps and side information via a multi-scale attention mechanism. The proposed MSPAConv, illustrated in Figure 2, consists of two key components:

*(1) Multi-parallel convolutional kernels*. We denote the kernels as $w_{i},i=1,2,\ldots ,k$. Such a design enables simultaneous processing of input features from diverse perspectives. When handling feature maps with varying texture patterns, different kernels capture distinct characteristics, thereby improving model capacity and flexibility.

*(2) Adaptive multi-scale weight estimation*. To achieve adaptive filtering with a fixed set of pre-trained parameters at a low computational cost, we introduce adaptive weights $a_{i}$ for each kernel $w_{i}$ and formulate the final convolutional parameters as a linearly weighted combination of the basis kernels:

(1) $$W=\sum_{i=1}^{k}a_{i}\cdot W_{i},$$

in which W represents the convolution parameter applied to the inputs, and $W_{i}$ denotes a learned parameter for the basis kernel $conv_{i}$.

The motivation behind the multi-scale design is to enable the filters to distinguish contents sharing similar global features but varying in local details. To estimate the adaptive weights $a_{i}\left(F_{\mathrm{in}}\right)$, we aggregate the input $F_{\mathrm{in}}$ using a pyramid pooling mechanism to perform multi-scale sampling, capturing spatial structural information across different scales. This enables the model to dynamically assign appropriate weights to each parallel kernel based on the input data at both global and local levels, thereby flexibly adjusting each kernel’s contribution to the final output. Specifically, the input is spatially average-pooled with different kernel sizes via adaptive average pooling (AAP). The pooled results are then reshaped and concatenated to form a multi-scale contextual representation

(2) $$F_{\mathrm{multi}-\mathrm{scale}}=\left\{S_{0}\left(F_{\mathrm{in}}\right);S_{1}\left(F_{\mathrm{in}}\right);\ldots ;S_{m}\left(F_{\mathrm{in}}\right)\right\},$$

in which $S_{m}\left(\cdot \right)$ refers to the operation of average-pooling $F_{\mathrm{in}}$ with an adaptive size. For a feature map $F_{\mathrm{in}}\in R^{c\times w\times h}$, the dimension of $S_{m}\left(F_{\mathrm{in}}\right)$ is $R^{c\times 2^{m}\times 2^{m}}$. Thus, the final dimension of $F_{\mathrm{multi}-\mathrm{scale}}$ is $c\sum_{j=0}^{m}2^{j}$. Afterward, $F_{\mathrm{multi}-\mathrm{scale}}$ is processed through a fully connected layer and a sigmoid layer to constrain its values within the range (0, 1). The overall pipeline is

(3) $$a_{i}\left(F_{\mathrm{in}}\right)=Sigmoid\left(FC\left(F_{\mathrm{multi}-\mathrm{scale}}\right)\right).$$

With the above-obtained kernels $Conv_{i}$ and adaptive weights $a_{i}\left(F_{\mathrm{in}}\right)$, the parameter-adaptive convolution can be formulated as

(4) $$F_{\mathrm{out}}=\left(\underset{\mathrm{Adaptive}\mathrm{Parameter}\;W}{\underset{︸}{\sum_{i=1}^{k}a_{i}\left(F_{\mathrm{in}}\right)\cdot W_{i}}}\right)*F_{\mathrm{in}}.$$

The results are then passed through a non-linear activation function. In our implementation, PReLU is adopted as the activation function, following the practice in most ILF networks.

### 3.2. S-MSPAConv: Side-Information-Modulated MSPAConv

Existing ILF methods typically utilize side information (SI) as auxiliary input to fully leverage intermediate encoding results. However, most networks simply concatenate SI with the reconstructed frames (rec) before passing them through a deep network, which often diminishes the contributive effectiveness of SI. To address this, we propose S-MSPAConv, which modulates side information into the weights of MSPAConv. This mechanism is formulated as follows:

(5) Fout=S−MSPAConv(Fin′)=MSPAConv(Fin;SI)=(∑i=1kai(Fin;SI)·Wi︸AdaptiveParameterW)∗∑i=1k{Fin;SI}︸Inputs,

where $F_{\mathrm{in}}^{\prime }$ denotes the input to S-MSPAConv and {·; ·} represents the concatenation operation. Below, we theoretically analyze how S-MSPAConv mitigates the aforementioned limitation by using the quantization parameter (QP) as a specific form of SI. Since a single model is typically required to handle all QPs, the QP map is frequently employed as side information. We first demonstrate that classical convolutional ILF networks only modulate the input QP via the bias term. Conversely, we show that S-MSPAConv extends this modulation to the convolutional parameters, thereby enabling more comprehensive feature adaptation.

(1) QP modulation in fixed-parameter networks. In classical ILF networks, the QP map is typically concatenated with the feature maps $F_{\mathrm{in}}$ and other side information ${\mathrm{SI}}_{o}$. This combined input is then passed through a convolution layer with fixed weights $W_{f}$ and bias $b_{f}$ to achieve QP adaptation, which can be formulated as follows:

(6) $$F_{\mathrm{out}}=W_{f}*\left\{F_{\mathrm{in}};\;{\mathrm{SI}}_{o};\;\mathrm{QP}\right\}+b_{f}.$$

Considering the convolution operation across different channels, this equation can be expanded as

(7) $$\begin{array}{cc} F_{\mathrm{out}} & =W_{f}^{\prime }*\left\{F_{\mathrm{in}};\;{\mathrm{SI}}_{o}\right\}+W_{f}^{\prime \prime }*QP+b_{f}\\ \; & =W_{f}^{\prime }*\left\{F_{\mathrm{in}};\;{\mathrm{SI}}_{o}\right\}+QP\cdot \sum W_{f}^{\prime \prime }+b_{f}. \end{array}$$

$W_{f}^{\prime }$ and $W_{f}^{\prime \prime }$ denote the weights applied to $\left\{F_{\mathrm{in}};\;{\mathrm{SI}}_{o}\right\}$ and QP, respectively. $\sum W_{f}^{\prime \prime }$ represents the sum of all parameters within the corresponding convolutional kernel. It is important to note that all elements within the QP channel are identical, as the QP value remains constant under common test conditions (CTC) when the adaptive QP for the coding-tree unit (CTU) is disabled. Therefore, the QP map can be separated from the weights in the second term of Equation (7), yielding $W_{f}^{\prime \prime }*QP=QP\cdot \sum W_{f}^{\prime \prime }$. It can be observed that concatenating the QP map as an input channel is mathematically equivalent to introducing a QP-dependent linear term into the bias. However, it is the weights, rather than the bias, that fundamentally determine the expressive capacity and performance of neural networks.

(2) QP modulation in S-MSPAConv. The proposed S-MSPAConv naturally addresses this limitation. By substituting SI with $\left\{{\mathrm{SI}}_{o};\;QP\right\}$ in Equation (5), we derive the following expression:

(8) Fout=(∑i=1kai(Fin, SI)·Wi︸AdaptiveParameterW)∗∑i=1k{Fin; SI}︸Inputs=W(Fin, SIo, QP)∗{Fin; SIo; QP}=W′(Fin, SIo, QP)∗{Fin; SIo}+QP·∑W″(Fin, SIo, QP),

where $W^{\prime }\left(F_{\mathrm{in}},\;{\mathrm{SI}}_{o},\;QP\right)$ and $W^{\prime \prime }\left(F_{\mathrm{in}},{\mathrm{SI}}_{o},QP\right)$ represent the parameters applied to $\left\{F_{\mathrm{in}};{\mathrm{SI}}_{o}\right\}$ and the QP map, respectively. It can be observed that in S-MSPAConv, the QP map is modulated not only within the bias term but also directly within the convolutional parameters. Consequently, S-MSPAConv provides superior effectiveness and robustness in terms of achieving QP adaptation.

### 3.3. Parameters and Complexity

The mathematical model demonstrates that S-MSPAConv and MSPAConv share the same structural logic, differing only in the input feature space. We introduce S-MSPAConv specifically to evaluate the impact of side information (SI) on the base MSPAConv operator, particularly regarding its advantages in QP-aware adaptation. While the integration of SI in S-MSPAConv enhances performance and adaptability, it also incurs additional parameter and computational costs. Thus, the choice between MSPAConv and S-MSPAConv reflects a trade-off between performance gains and computational efficiency.

Table 1 compares the computational complexity and parameter counts among a regular convolution, the proposed MSPAConv, and S-MSPAConv. Compared with a regular convolution, MSPAConv retains similar complexity due to its linearly weighted parameter structure. However, it introduces a moderate increase in parameters, which depends on the convolutional kernels and the fully connected (FC) weights shown in Figure 2. The former is proportional to *k*, while the latter is proportional to the product of the input dimension $c\sum_{j=0}^{m}2^{j}$ and the output dimension *k* of the FC layer. For S-MSPAConv, since SI is concatenated along the channel dimension of $F_{\mathrm{in}}$, the complexity increases by a factor of $\frac{l}{c}$, where *l* denotes the channel dimension of SI and *c* is the original channel dimension of $F_{\mathrm{in}}$. The total parameter count remains consistent with MSPAConv. Consequently, the proposed MSPAConv and S-MSPAConv should not naively replace standard convolutions in existing in-loop filtering networks without considering these overheads. In our implementation, we empirically determine the hyper-parameter *k* via experimental results. Furthermore, we employ only the QP map and image gradients as additional side information for S-MSPAConv, as the QP map serves as a critical factor for global noise estimation, while gradients capture image details, forcing the network to focus more on local variations.

## 4. PAILF-Net: Parameter-Adaptive In-Loop Filtering Network

To address the limitations of fixed-weight paradigms in existing neural network-based in-loop filtering methods, this section proposes a parameter-adaptive ILF network (PAILF-Net) leveraging our MSPAConv and S-MSPAConv modules. We first present an overview of the overall pipeline of PAILF-Net, followed by a detailed description of its four primary modules.

### 4.1. The Overall Pipeline

**Figure 3:**
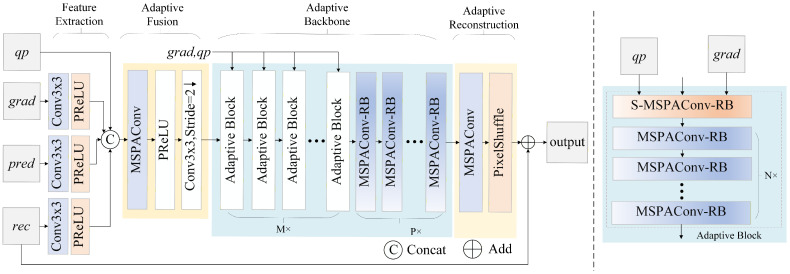
The proposed PAILF-Nets. rec, pred, grad, and qp denote the input tensors with 3, 3, 3, and 1 channels, respectively. The output channel for all 3×3 convolutions in the feature-extraction module is 64. In the adaptive fusion module, the kernel size and output channel count of MSPAConv are 1×1 and 64, respectively. The MSPAConv-RB and S-MSPAConv-RB blocks are illustrated in Figure 4b and Figure 4c, respectively. In our implementation, *M*, *N*, and *P* are set to 5, 5, and 2, respectively, to maintain a fair comparison with the baseline JVET-AC0194. The strides for all convolution kernels are set to 1, unless otherwise specified.

Based on the proposed MSPAConv and S-MSPAConv modules, PAILF-Net is designed to adapt to diverse image contents and varying degrees of compression artifacts. In contrast to traditional fixed-parameter convolutional architectures, our parameter-adaptive mechanism enhances the network’s adaptability, enabling it to handle complex coding distortions more effectively and thereby improving overall coding efficiency. Figure 3 illustrates the overall framework of PAILF-Net. The structure is similar to Tencent’s residual network [41], which has been widely adopted in recent JVET proposals for ILF, e.g., [34,42]. The primary novelty lies in the integration of our parameter-adaptive mechanism and edge-aware side information grad. PAILF-Net primarily comprises four parts: (1) feature extraction; (2) adaptive fusion; (3) adaptive backbone; and (4) adaptive reconstruction.

**Figure 4:**
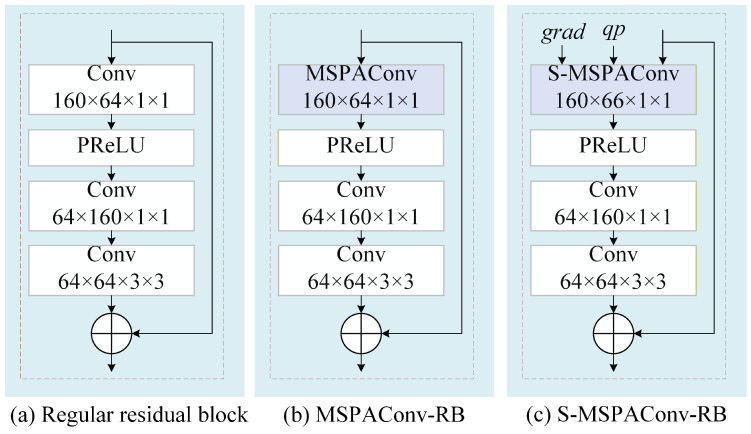
Structures of the regular residual block, MSPAConv-RB, and S-MSPAConv-RB. The kernel sizes for both MSPAConv and S-MSPAConv are set to 1×1, and the strides for all convolutional layers are set to 1.

Given the to-be-filtered reconstruction rec and the side information, i.e., the prediction pred and gradient grad, the feature-extraction module (Section 4.2) first extracts features from each source for efficient filtering. The results are then concatenated with the QP map qp and fused via adaptive fusion (Section 4.3), allowing complementary information to be integrated. Subsequently, the adaptive backbone and reconstruction module (Section 4.4) performs adaptive filtering and reconstruction, ultimately outputting high-quality restored images.

### 4.2. Feature Extraction

Similar to [41], the inputs of the proposed network include the reconstructed frame rec, the generated prediction frame pred during VVC encoding, and the QP map qp. Since rec and pred are in the YUV420 domain, we upsample the U and V components to match the resolution of the Y, unifying them with one model. Given that the primary objective of ILF is to restore quantized details, operating directly on pixel values in the reconstruction domain may inadvertently cause the network to prioritize absolute intensity values over structural differences. To mitigate this, we adopt the gradient grad of the Y-component reconstruction $rec_{y}$ as a guidance signal to capture edges and emphasize local discontinuities between neighboring pixels. The gradient grad(x, y) at position (x, y) is computed as

(9) $$grad\left(x,\;y\right)=\sqrt{rec_{y_{x}}^{2}\left(x,\;y\right)+rec_{y_{y}}^{2}\left(x,\;y\right)},$$

where $rec_{y_{x}}\left(x,\;y\right)=rec_{y}\left(x+1,\;y\right)-rec_{y}\left(x-1,\;y\right)$ and $rec_{y_{y}}\left(x,\;y\right)=rec_{y}\left(x,\;y+1\right)-rec_{y}\left(x-1,\;y-1\right)$ represent the horizontal and vertical gradients at position (x, y), respectively. Subsequently, rec, pred, and grad are passed through the feature-extraction module, where each source undergoes a dedicated convolution followed by a non-linear activation function. The resulting features are concatenated with the qp map to generate the comprehensive input representation for the subsequent network

(10) $$f_{\mathrm{in}}=concat\left(f_{\mathrm{rec}},\;f_{\mathrm{pred}},\;f_{\mathrm{grad}},\;\mathrm{qp}\right).$$

### 4.3. Adaptive Fusion

Following the concatenation, the features are fed to the fusion network. Since the fusion stage occurs in the earlier stage of the network and SI, i.e., qp, pred, and grad, is already implicitly or explicitly text-color-blue integrated into the fused input $f_{\mathrm{in}}$, we adopt the MSPAConv operator to perform parameter-adaptive fusion. This process is formulated as

(11) $$f_{\mathrm{fuse}}=MSPAConv\left(f_{\mathrm{in}}\right).$$

By combining Equations (10) and (11), we demonstrate that this fusion process is mathematically equivalent to S-MSPAConv, where the side information SI is defined as $\left\{f_{\mathrm{pred}};\;f_{\mathrm{grad}};\;\mathrm{qp}\right\}$. Subsequently, the fused feature maps undergo non-linear activation via PReLU. To reduce computational complexity in subsequent stages, a 3×3 convolution with a stride of 2 is employed to perform feature downsampling, a design choice consistent with most state-of-the-art in-loop filters.

### 4.4. Adaptive Backbone and Reconstruction

Diverse image content often results in distinct structural distortions, such as ringing artifacts in complex texture areas and blocking effects in smooth regions. To mitigate these distortions, conventional ILF networks employ brute-force strategies, such as increasing network depth, expanding width, or incorporating sophisticated architectures like transformers. While these approaches can incrementally improve model performance, they invariably impose a significant computational burden. Conversely, we introduce a parameter-adaptive mechanism through the proposed MSPAConv and S-MSPAConv modules, which effectively enhances model capacity with negligible increases in computational overhead.

Specifically, we utilized the baseline architecture from JVET-AC0194, which is primarily composed of convolutional residual blocks. To facilitate adaptivity, we designed two specialized parameter-adaptive residual blocks: MSPAConv residual blocks (MSPAConv-RB) and S-MSPAConv residual blocks (S-MSPAConv-RB). Figure 4a–c illustrate the detailed structures of the residual blocks utilizing regular convolution, MSPAConv, and S-MSPAConv, respectively. Compared with the regular residual block, MSPAConv-RB replaces the initial convolution layer with MSPAConv to capture adaptive features at the earliest possible stage while constraining parameter growth. S-MSPAConv-RB further integrates side information to provide enhanced guidance throughout the intermediate layers. Given that the computational complexity of S-MSPAConv scales with the number of stacked SI channels, we selectively incorporate only grad and qp as SI, where grad encodes textural details and qp reflects the characteristics of compression-induced distortions.

The adaptive backbone comprises *M* adaptive blocks followed by *P* tailing MSPAConv-RBs. Each adaptive block consists of one S-MSPAConv-RB interleaved with *N* MSPAConv-RBs to maintain a balanced trade-off between performance and computational complexity. The tailing MSPAConv-RBs are incorporated to ensure a fair comparison with the baseline architecture, compensating for cases where the total number of residual blocks is not perfectly divisible by N+1. We denote the final output features of this stage as $f_{\mathrm{out}}$.

### 4.5. Adaptive Reconstruction

Finally, the feature representation $f_{\mathrm{out}}$ is further enhanced via a single MSPAConv-RB and subsequently upsampled through a PixelShuffle layer. The final filtered reconstruction is expressed as follows:

(12) $$r\hat{e}c=\mathrm{rec}+PixelShuffle\left(MSPAConv\left(f_{\mathrm{out}}\right)\right).\;$$

rec denotes the input reconstructed frame containing the Y component and the upsampled UV components, while $r\hat{e}c$ represents the final restored output of the network.

## 5. Experimental Results

This section is organized as follows: first, we detail the experimental configurations; next, we present the comprehensive results; and, finally, we perform ablation studies, encoding time and memory usage evaluation.

### 5.1. Experimental Settings

#### 5.1.1. Dataset and Training Setting

The DIV2K [43] dataset is utilized to train our proposed filter, employing the 800 training images at 2K resolution. Training data is generated by compressing these images using the VTM-11.0 reference software with all in-loop filters disabled. We adopt quantization parameters (QPs) of {22, 27, 32, 37, 42}, resulting in a total of 4000 training samples. These compressed images are randomly cropped into 144×144 patches. Thus, the total number of patches depends on the batch size and training epochs. Data augmentation, including random rotation and horizontal flipping, is applied to each epoch during the training process.

The proposed network is implemented in PyTorch 1.11.0. The initial learning rate is set to $10^{-4}$, which is halved every $6\times 10^{4}$ iterations, with a lower bound of $1.25\times 10^{-5}$. The model is trained for a total of 160,000 iterations with a batch size of 16. The training procedure is conducted on a server equipped with an Intel Core i7-14700F CPU and dual NVIDIA GeForce RTX 4090 GPUs, requiring approximately 3.5–4 days of training time. The mean absolute error (MAE) is initially utilized as the loss function to stabilize training, followed by the mean squared error (MSE) to fine-tune convergence after 80,000 iterations. The loss weights for the Y, U, and V components are set to 10:1:1, respectively. The Adam [44] optimizer is employed for network optimization.

#### 5.1.2. Implementation

In our implementation, we almost keep the original architectural parameters of our baseline, i.e., JVET-AC0194, for fair comparison, except that partial dimensions are required to match with the proposed S-MSPAConv. For a fair comparison with the baseline JVET-AC0194, *M*, *N*, and *P* are set to 5, 5, and 2, respectively. *k* is set to 4 to make a better trade-off between performance and parameters. After training, the network is integrated into the VVC reference software VTM-11.0. As shown in Figure 1c, the proposed filter network is used to replace DBF and SAO under all intra (AI) configurations. The rate-distortion optimization process is employed to select the optimal filtered outcome between traditional filtering and the proposed method. Additionally, the proposed filter network can be adaptively enabled or disabled at the coding-tree-unit level and slice level.

In our implementation, we maintain the architectural hyperparameters of the baseline model JVET-AC0194 to ensure a fair comparison, adjusting partial dimensions solely to align with the S-MSPAConv requirements. To further ensure consistency, the parameters *M*, *N*, and *P* are set to 5, 5, and 2, respectively, while *k* is set to 4 to optimize the trade-off between coding performance and model parameters. Following training, the network is integrated into the VVC reference software VTM-11.0. As illustrated in Figure 1c, the proposed filter network replaces the DBF and SAO under all-intra (AI) configurations. The rate-distortion optimization (RDO) process is employed to select the optimal filtered outcome between the traditional filtering approach and our proposed method. Furthermore, the proposed filter network can be adaptively enabled or disabled at both the CTU and slice levels, providing flexible control over the filtering process.

#### 5.1.3. Compared Methods

We categorize the compared approaches into two classes based on their parameter adaptation mechanisms:

(1) Fixed-parameter networks. This category includes methods where the network weights remain fixed during inference, regardless of the input content or coding conditions:

- QA-Filter [45]: Specifically addresses quantization parameter (QP) adaptation, enhancing filtering performance through optimized QP-based processing.
- SwinIR [20]: Implements in-loop filtering by leveraging the hierarchical Swin transformer architecture.
- JVET-AA0088 [33] and JVET-AC0194 [34]: State-of-the-art neural network-based in-loop filtering proposals presented in recent JVET meetings.
- Baseline of Meta-ILF: The fixed-parameter version of the Meta-ILF framework, serving as a control.
- DILF [23]: a dynamic neural network approach for in-loop filtering that handles both QP and content adaptation. Unlike our method which dynamically adjusts model parameters, DILF achieves adaptation via dynamic network structure modifications.

(2) Adaptive parameter networks. This category includes methods that dynamically adjust network parameters to better align with specific image content or distortions:

- Meta-ILF [37]: Specifically designed for image-content adaptation, though its original design focuses exclusively on optimizing the luma component.

For the QA-Filter, Meta-ILF and its corresponding baselines, SwinIR, JVET-AA0088, and DILF, we directly adopt the results reported in their original publications. For the proposed PAILF-Net, we designate JVET-AC0194 as the baseline framework. To ensure a rigorous and fair comparison, we maintain identical training configurations between JVET-AC0194 and our method, ensuring that performance differences are attributable solely to the network architecture.

All compared methods employ VTM-11.0 as the reference anchor. The experimental settings strictly conform to the common test conditions (CTC) [46] defined by JVET. We utilize standard test sequences, i.e., Class A1, A2, B, C, and E, as the test set and evaluate performance using the Bjøntegaard-delta rate (BD-rate) [47], with the peak signal-to-noise ratio (PSNR) serving as the primary quality metric. To calculate the weighted average BD-rate, we apply a ratio of 6:1:1 to the Y, U, and V components, respectively. Five QPs are tested: {22, 27, 32, 37, 42}. Computational complexity is evaluated in terms of kilo-multiply-accumulate operations (kMACs) and the total count of network parameters.

### 5.2. Comparison with State-of-the-Art

The performance evaluation of the proposed PAILF-Net is summarized in Table 2. As demonstrated, PAILF-Net achieves an average BD-rate reduction of 10.59%, with a component-specific BD rate of −7.89%, −18.25%, and −19.15% for the Y, U, and V components, respectively. Compared with the baseline JVET-AC0194, the integration of our parameter-adaptive mechanism enables PAILF-Net to realize an additional bitrate reduction of 0.77%, 3.31%, and 3.43% for Y, U, and V, respectively, while maintaining comparable computational complexity. It also significantly outperforms JVET-AA0088. When benchmarked against Meta-ILF, our approach proves superior, as Meta-ILF’s content-adaptive algorithm yields only a marginal 0.4% gain over its baseline. Furthermore, relative to the QP-adaptive QA-Filter, our method exhibits substantial performance advantages, with gains of 2.76%, 13.70%, and 13.82% for the Y, U, and V components. Additionally, PAILF-Net outperforms the transformer-based SwinIR in terms of both coding efficiency and computational overhead. Although DILF exhibits superior BD-rate and complexity profiles, it primarily addresses adaptation via dynamic architectural modifications rather than parameter adaptation. Consequently, integrating our proposed parameter-adaptive approach with DILF will further improve its performance.

Beyond coding performance, PAILF-Net requires only a negligible computational overhead of approximately 1 kMACs/pixel, relative to the 485 kMACs/pixel baseline of JVET-AC0194. This result aligns with our theoretical analysis in Section 3.3, confirming an optimal trade-off between coding efficiency and computational complexity. While Meta-ILF attempts to reduce complexity, its adaptive mechanism substantially inflates the model size from 1.4 M to 5.4 M parameters. In contrast, our proposed method requires an additional 1.6 M parameters, which is significantly more resource-efficient than the 5.4 M parameters required by Meta-ILF or the 10.9 M parameters utilized by the transformer-based SwinIR. Furthermore, PAILF-Net achieves superior rate-distortion performance while maintaining lower computational costs. In summary, PAILF-Net enhances video-coding efficiency with negligible increase in complexity, demonstrating its practicability and architectural superiority for real-world video-coding applications.

Table 3 details the comparative performance against the JVET-AC0194 baseline. As demonstrated, PAILF-Net consistently enhances coding performance across all test classes. The most significant gains are observed in class A2, with reductions of 0.93%, 5.33%, and 7.63% for the Y, U, and V components, respectively. While the reduction in the Y component for Class E is relatively more modest at 0.67%, the U and V components achieve notable gains of 5.32% and 4.19%. PAILF-Net consistently provides improvements on Y component, with rate savings ranging from 0.42% to 1.22%. Notably, for the chroma component, the enhanced kernels and our adaptive mechanism allows for more specialized processing compared to the Y component. The performance gains reach up to 11.87% and 11.06% for the U and V components, respectively.

**Figure 5:**
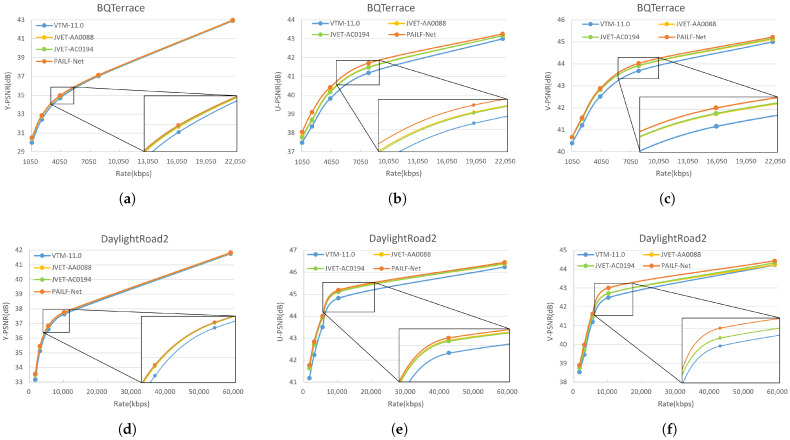

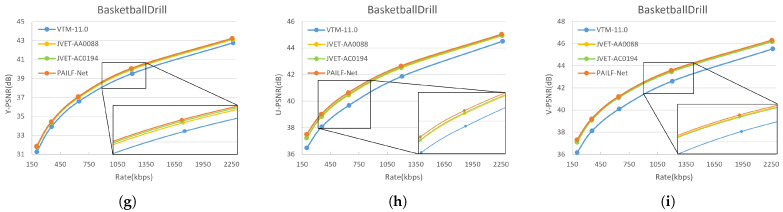
The RD curve comparisons across different sequences and components. (**a**–**i**) illustrate the Y-PSNR, U-PSNR, and V-PSNR versus bitrate curves for the BQTerrace, DaylightRoad2, and BasketballDrill sequences, respectively.

Figure 5 illustrates the RD performance of the Y, U, and V components for the BQTerrace, DaylightRoad2, and BasketballDrill. It is evident that the proposed PAILF-Net consistently outperforms both JVET-AC0194 and JVET-AA0088 across all tested bitrates for these sequences. Specifically, for the BQTerrace sequence, PAILF-Net achieves a peak gain of 0.44 dB for the U component at a bitrate of 2091 kbps relative to the JVET-AC0194 baseline. Furthermore, for the V component, our method realizes improvements of up to 0.29 dB at bitrates of 10,140 kbps and 3031 kbps. While performance gains for the Y component are also evident, they are relatively more modest compared to the substantial improvements observed in the U and V components.

To verify the effectiveness of the proposed PAILF-Net, we evaluate its performance under random access (RA) and lowdelay B (LDB) configurations. Due to the high computational complexity of the evaluation process, class B and C sequences are utilized for RA, while class B, C, and E are used for LDB. For each sequence, only the first 64 frames are compressed. Table 4 and Table 5 summarize the comparative results against the JVET-AC0194 baseline for the RA and LDB configurations, respectively. As observed in the RA configuration, PAILF-Net demonstrates consistent improvements across all evaluated classes, achieving bitrate savings of {0.56%, 6.46%, 3.05%} for class B and {0.43%, 2.98%, 2.87%} for class C across the {Y, U, V} components, respectively. The average bitrate reduction reaches {0.49%, 4.52%, 2.95%}. In the LDB configuration, PAILF-Net also exhibits performance gains across all test classes. Specifically, bitrate savings of {0.55%, 5.29%, 2.32%}, {0.53%, 2.03%, 2.82%}, and {0.63%, 6.62%, 4.82%} are obtained for class B, C, and E, respectively, resulting in an average saving of {0.56%, 4.54%, 3.11%}. These results under both RA and LDB configurations further validate the superior coding performance of the proposed PAILF-Net.

### 5.3. Qualitative Evaluation

**Figure 6:**
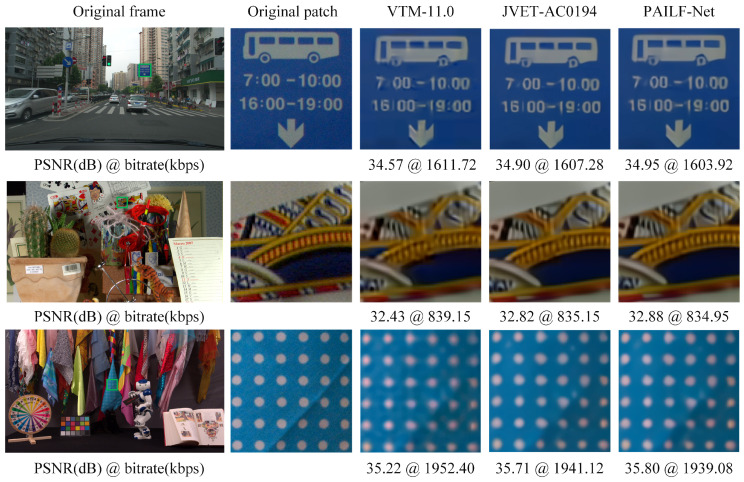
Subjective quality comparison across different sequences. The rows, from top to bottom, correspond to the first frames of the DaylightRoad2, Cactus, and CatRobot under the AI configuration with QP=42. The columns represent: (1) original video frames; (2) uncompressed image patches corresponding to the green region; (3) compressed image of VTM-11.0 with traditional filters; (4) compressed image of JVET-AC0194; and (5) compressed image of the proposed PAILF-Net.

Figure 6 shows a visual quality comparison among VTM-11.0, JVET-AC0194, and the proposed PAILF-Net. We present three visual examples using the first frames of DaylightRoad2, Cactus, and CatRobot, encoded at *QP* = 42. It is evident that the VTM-11.0 reconstruction in the first row exhibits distinct artifacts around the digits “7” and “16,” which severely degrade perceptual quality. Although the application of JVET-AC0194 mitigates these artifacts, residual degradation persists. In contrast, our proposed method demonstrates superior subjective quality. In the second row, PAILF-Net produces sharper and more defined edges on the playing cards, whereas the VTM-11.0 and JVET-AC0194 results appear noticeably blurred. In the third row, the proposed method effectively reconstructs the “white dots” with significantly reduced artifacts compared to the baseline methods. These visual results consistently demonstrate that our adaptive filtering mechanism provides enhanced subjective quality, further verifying its efficacy in improving overall video coding performance. While PAILF-Net significantly enhances visual fidelity, we acknowledge that in-loop filtering alone cannot fully eliminate all artifacts introduced by heavy quantization during the encoding process.

### 5.4. Ablation Studies

Overview and Interaction of Key Improvements. The proposed PAILF-Net enhances coding performance primarily through the integration of a parameter-adaptive mechanism. Although interactions may exist between different components, an exhaustive evaluation of all combinations is computationally prohibitive. Given that the overall improvement of PAILF-Net is moderate, the synergistic gains from combining various components are expected to remain within a bounded range. Consequently, we focus on an ablation study by systematically removing each component to evaluate its contribution. Table 6 summarizes the performance gains attributed to each core module, namely the multi-scale adaptive parameter mechanism, the QP integration in S-MSPAConv, and the grad feature in S-MSPAConv. The first row represents the full PAILF-Net configuration. Subsequent rows present the results as key components are progressively removed, with the final row corresponding to the JVET-AC0194 baseline. It can be observed that the integration of gradient features provides gains of {0.16%, 0.96%, 1.49%}. The removal of both gradient and QP-based features leads to a cumulative performance loss of {0.3%, 0.82%, 1.3%}. Furthermore, the exclusion of the multi-scale adaptive parameter mechanism yields a significant BD-rate degradation, with performance dropping from {−7.59%, −17.43%, −17.85%} to {−7.12%, −14.94%, −15.72%}. This constitutes an additional bitrate loss of {0.47%, 2.49%, 2.13%} for the Y, U, and V components, respectively. These results indicate that while the multi-scale adaptive parameter mechanism is the primary contributor to coding efficiency, the remaining modules provide essential and complementary gains. The following sections provide a detailed analysis of each component.

The parameter adaptive mechanism in each module. To validate the effectiveness of the proposed parameter-adaptive mechanism across each module, i.e., the feature extraction, adaptive backbone, and adaptive reconstruction modules, we conduct two sets of comparative experiments.

The first set is presented in the second row of Table 7, where the MSPAConv and S-MSPAConv units in the feature extraction and adaptive reconstruction modules are replaced with conventional 3×3 convolutions. The experimental results indicate that the BD-rate performance degrades from {−7.89%, −18.25%, −19.15%} to {−7.68%, −17.03%, −18.08%}, corresponding to a 0.21%, 1.22%, and 1.07% performance loss in the Y, U, and V components, respectively. This demonstrates that regular convolutions lack adaptive adjustment capabilities, impeding flexible adaptation of extraction and reconstruction strategies. In contrast, MSPAConv and S-MSPAConv dynamically adjust convolution-kernel parameters based on quantization parameters and local image content, thereby flexibly modulating filter strength for improved adaptation.

The final row in Table 7 replaces the MSPAConv-RB and S-MSPAConv-RB in the adaptive backbone module of Figure 3 with the regular residual block depicted in Figure 4a. The results show that compared with the full PAILF-Net, the absence of MSPAConv-RB and S-MSPAConv-RB leads to a decline in the average BD rate from −10.59% to −9.67%, with 0.54%, 2.01%, and 2.1% bitrate increases for the Y, U, and V components, respectively. The network backbone must process the diverse image features that standard residual blocks struggle to effectively characterize. Conversely, our proposed adaptive parameter mechanism adjusts convolution-kernel parameters according to image content, enhancing feature extraction capacity. By utilizing quantization parameters and gradient information to guide network optimization, our approach improves the model’s overall capability.

**Figure 7:**
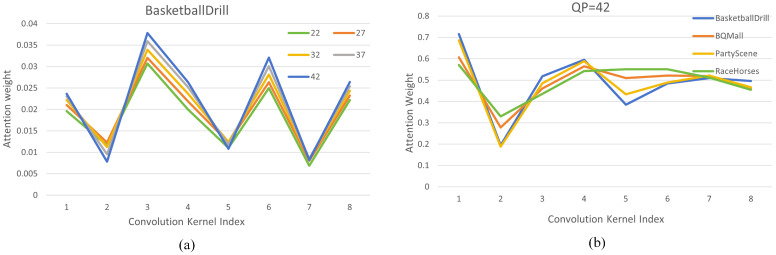
Visualization of the attention-weight distribution. (**a**) Distributions in the final layer across varying encoding QPs for BasketballDrill. (**b**) Distributions in the initial layer across different video sequences under a consistent encoding QP.

Visualization of the learned paralleled weights. To reveal the adaptive nature of the proposed MSPAConv, we set k=8 and visualize the attention-weight distribution of one kernel. Regarding QP adaptation, Figure 7a illustrates the attention-weight distribution for BasketballDrill across different encoding QP values. It is evident that when the QP is 42, the weights of all parallel convolution kernels are at a higher magnitude compared to those at lower QP values. This can be interpreted as follows: as the QP value increases, the resulting reconstruction distortion becomes more severe. To mitigate this, a stronger filtering intensity is required. The proposed PAILF-Net effectively captures the distortion level corresponding to different QP values and dynamically modulates the convolution kernel weights accordingly, demonstrating superior image restoration and adaptive adjustment capabilities.

For content adaptation, Figure 7b displays the attention weight distribution for different video sequences at QP=42. It is clearly observable that different video sequences exhibit distinct weight distribution patterns. Given the diversity of content feature, e.g., scene characteristics, texture complexity, and chromatic richness, the distortion patterns vary significantly, necessitating tailored adaptation. The observed variations in weight distribution among different videos confirm that the proposed network can adaptively select optimal convolution kernels based on local image content to extract relevant features.

Multi-scale within MSPAConv and S-MSPAConv. To investigate the impact of the multi-scale mechanism within MSPAConv and S-MSPAConv on parameter adaptation, this section evaluates a simplified approach to generating attention weights for parallel convolution kernels by utilizing only the AAP(1) configuration, as illustrated in Figure 2. Specifically, global average pooling is first applied to the input features to aggregate spatial information, which is then processed by a fully connected (FC) layer. Finally, the FC layer’s outputs are transformed into attention weights for parallel kernels via a Sigmoid activation function. This simplified parameter-adaptive convolution is applied to our proposed PAILF-Net, and the experimental result is denoted as “w.o. multi-scale” in Table 8. Compared with the full PAILF-Net architecture, it exhibits a performance decrease of 0.16%, 1.11%, and 1.09% for the Y, U, and V components, respectively. The analysis underscores the advantages of the multi-scale mechanism: at larger scales, i.e., smaller AAP sizes, kernels focus on global structural patterns, while at finer scales, they specialize in local texture characteristics. This hierarchical approach allows the generated kernel parameters to adapt effectively to diverse image contents and varying distortion levels, ultimately yielding more accurate reconstruction.

The number of paralleled kernels (k). To evaluate the impact of increasing the number of parallel convolution kernels *k* in MSPAConv and S-MSPAConv, we conduct an experiment with k=8 for the proposed PAILF-Net. The results are presented in the columns denoted as “PAILF-Net (k=8)” in Table 8. Compared with the PAILF-Net configuration using the default k=4, the averaged BD rate improves from {−7.89%, −18.25%, −19.15%} to {−8.19%, −19.22%, −20.34%}, yielding additional rate reduction of 0.3%, 0.97%, and 1.29% for the Y, U, and V components, respectively. These findings clearly demonstrate that increasing the number of parallel kernels enhances performance in both luma and chroma components without incurring additional computational overhead during inference, thereby highlighting a superior adaptability profile without sacrificing computational efficiency. Nevertheless, the model parameters increase significantly by more than 55%, growing from 3.1 M to 4.8 M. This expansion necessitates extended training durations and hyperparameter optimization to mitigate the risk of converging to local optima.

The impact of gradient (grad). To validate the effectiveness of the gradient map (grad), we conduct an ablation study by removing grad from the PAILF-Net architecture. In this configuration, grad is neither extracted from the reconstructed images nor fed into the network. Consequently, within the adaptive backbone, only the quantization parameter map (qp) is utilized to guide the learning of S-MSPAConv. The experimental results are presented in the columns denoted as “w.o. grad” in Table 9. Compared with the full PAILF-Net, the gradient-free approach exhibits a consistent performance degradation across all Y, U, and V components. Specifically, the averaged BD-rate performance decreases from {−7.89%, −18.25%, −19.15%} to {−7.73%, −17.29%, −17.66%}, representing a performance loss of 0.16%, 0.96%, and 1.49% for the Y, U, and V components, respectively. These results empirically confirm the contribution of gradient maps as a structural prior in enhancing the network’s feature representation and overall coding efficiency. We also investigated the use of the $L_{1}$ norm for gradient evaluation, specifically by defining $grad\left(x,\;y\right)=|rec_{x}|+|rec_{y}|$ as a replacement for the formulation in Equation (9). However, this approach failed to achieve convergence during training. The primary reason for this instability may be that the $L_{1}$ norm is non-differentiable at the origin, which hampers the backpropagation process in the proposed neural-network architecture.

**Figure 8:**
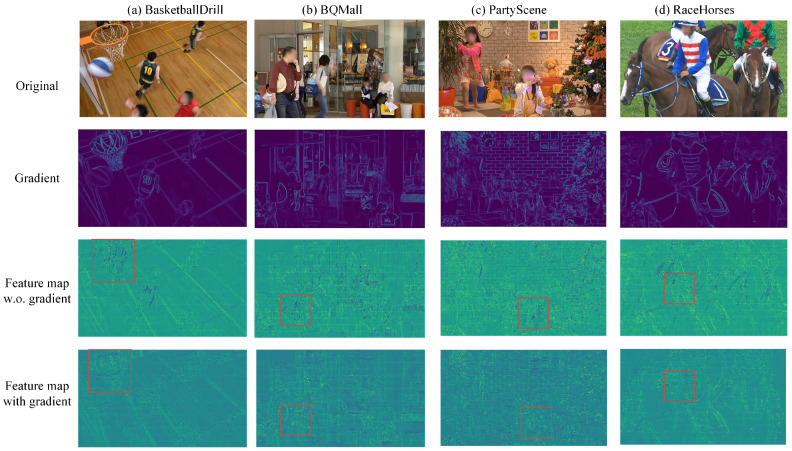
Visualization of gradients on feature maps. The first and second rows display the original intra frame and the luma reconstruction gradient at QP=42, respectively. The third and fourth rows show the output feature maps of the final residual block in the adaptive backbone, respectively, with and without the gradient map as side information in PAILF-Net. Due to privacy and copyright considerations, facial regions of the original frame in the first line have been blurred.

To further understand the significance of gradient maps in improving the perceptual quality of reconstructed images, Figure 8 visualizes feature maps with and without the integration of gradient priors. We select four representative video sequences: BasketballDrill, BQMall, PartyScene, and RaceHorses. The gradient maps of the first reconstructed frames are visualized in the second row of Figure 8. These maps effectively show texture details, e.g., the athletes’ movements in BasketballDrill and the pedestrians in BQMall. Furthermore, block artifacts arising from the encoding process appear as distinct, abnormal gradient discontinuities, e.g., the regular block-shaped contours visible in PartyScene.

We also visualize the output feature maps from the final residual block in the third and fourth rows of Figure 8 to intuitively demonstrate the impact of incorporating gradient priors. Specifically, the third and fourth rows show the activation patterns of the first frame for the four sequences before and after incorporating the gradient map into the network backbone, respectively. It is clearly observable that the images in the fourth row appear perceptually sharper, with enhanced preservation of fine-grained texture details compared to the third row. By leveraging gradient information, the network more accurately recovers edge and detail features, substantially enhancing subjective image quality. This comparison empirically demonstrates the effectiveness of utilizing gradient maps as a structural prior within the network.

The impact of QP qp within S-MSPAConv. To investigate the effectiveness of quantization parameters in guiding network learning, we further conducted an ablation study by removing the qp map from S-MSPAConv in the backbone, while retaining the global QP qp as an input to the entire network for a fair comparison, i.e., neither the gradient map nor the quantization parameter map is introduced into the backbone. The remaining experimental settings are kept consistent with those of PAILF-Net. The results are shown in the columns denoted as “w.o. grad and qp” of Table 9. It can be observed that, compared with the PAILF-Net “w.o. grad” configuration, the BD rate varies from {−7.73%, −17.29%, −17.66%} to {−7.59%, −17.43%, −17.85%} for the Y, U, and V components, respectively. Specifically, the integration of the QP map qp within S-MSPAConv leads to a 0.14% improvement for the Y component, while resulting in a 0.14% and 0.19% performance loss for the U and V components, respectively. Considering that the luma component is more prioritized than the chroma components in visual coding, we justify the utilization of qp as a guidance mechanism for S-MSPAConv.

### 5.5. Encoding Time and Memory Usage

Although we have analyzed the theoretical complexity and parameter count in Section 3.3, we also evaluate the encoding time and memory usage on the CPU to bridge the gap regarding practical application. For efficiency, we encode 10 frames for each sequence using {22, 27, 32, 37, 42} QPs. The time measurement methodology follows the CTC. The memory usage is sampled every two seconds for each encoding pass, and the results are subsequently averaged. The final time and memory metrics for each class are geometrically averaged, consistent with CTC. Table 10 shows the evaluated results, where $Ratio=\frac{\mathrm{PAILF}\text{-}\mathrm{Net}}{\mathrm{JVET}\text{-}\mathrm{AC}0194}$. It is observed that the baseline JVET-AC0194 requires an average of 1301.00 seconds and 1138.04 MB of memory for encoding. The proposed PAILF-Net requires 1351.07 seconds and 1365.15 MB of memory. Compared to JVET-AC0194, PAILF-Net exhibits a ratio of 1.04× and 1.20× for time and memory, respectively. This indicates that the proposed method incurs negligible computational overhead, and the memory consumption attributable to the increased parameter count remains well within acceptable limits for practical deployment. Furthermore, by comparing class A1 and A2, we observe that encoding time varies even for sequences of the same resolution, reflecting the impact of differing bitrates under identical QP settings.

## 6. Discussion

The experimental results demonstrate that the proposed adaptive mechanism achieves superior performance compared to fixed-parameter networks in all intra, random access and lowdelay B coding configurations and the tested sequences. However, our current strategy cannot theoretically guarantee superior performance across all sequences and coding settings, as learned adaptive weights may occasionally be outperformed by globally optimized fixed weights. To ensure robust performance, one future direction involves integrating a weight-fusion stabilization strategy:

(1) Pre-trained weights from fixed networks can be integrated into our proposed architecture as a baseline. For example, these weights can be assigned to any $Conv_{i}$ in the corresponding layer of the proposed adaptive network and subsequently frozen during training, thereby ensuring that the static-parameter design serves as a special case of our adaptive framework.

(2) By enabling a manually controlled weight-fusion mechanism via regulating the adaptive weights $a_{i}$, we can ensure that the adaptive network’s performance is at least as reliable as, and typically superior to, conventional fixed-parameter filters.

## 7. Conclusions

In this paper, we propose a parameter-adaptive neural in-loop filtering network (PAILF-Net) to address the parameter adaptation challenge for large-scale models. The core contributions are the proposed multi-scale parameter-adaptive convolution (MSPAConv) and its extension, side-information-modulated MSPAConv (S-MSPAConv), where the convolution parameters are modeled as a linearly weighted combination of pre-trained kernels, with weights estimated by input-dependent attention. Additionally, gradient maps are incorporated to enhance distortion-aware guidance. Experiments demonstrate that our adaptive mechanism improves reconstruction quality with negligible computational overhead.

While our current study focuses on the efficiency of parameter adaptation within a controlled architectural scale, an interesting area for future investigation is the relationship between the adaptive mechanism and network depth. We believe this exploration could further reveal the scalability of our approach across varying levels of model complexity. Future work will also include developing adaptive policies to more efficiently control the number of parallel kernels and extending the framework to small-scale models.

## Figures and Tables

**Table 1:** Comparison of parameter numbers and computational complexity among regular convolution, MSPAConv, and S-MSPAConv. Here, $P_{ara}$, *C*, and *c* represent the number of parameters, computational complexity, and number of channels for a regular convolution, respectively. The variable *k* denotes the number of paralleled kernels in both MSPAConv and S-MSPAConv, while *l* corresponds to the channel dimension of the side information incorporated in S-MSPAConv.

	Regular Convolution	MSPAConv	S-MSPAConv
Complexity	O(C)	O(C)	$O(\left(1+\frac{l}{c}\right)\cdot C)$
#Parameters	$P_{ara}$	$k\cdot P_{ara}+k\cdot c\sum_{j=0}^{m}2^{j}$	$k\cdot P_{ara}+k\cdot c\sum_{j=0}^{m}2^{j}$

**Table 2:** BD-rate (%) performance comparison of the proposed filters against state-of-the-art methods under the AI configuration. Negative values denote bitrate savings, indicating superior coding efficiency.

Type of Parameters	Approaches	BD-Rate				#Parameters	Complexity
		Y	U	V	Average	(M)	(kMACs/Pixel)
Fixed	QA-Filter [45] ^(2022)^	−5.13%	−4.55%	−5.33%	−5.08%	-	-
	SwinIR [20] ^(2024)^	−6.77%	−14.67%	−14.97%	−8.78%	10.9	11,384
	JVET-AA0088 [33] ^(2022)^	−6.50%	−14.88%	−15.97%	−8.73%	1.9	485
	JVET-AC0194 [34] ^(2023)^	−7.12%	−14.94%	−15.72%	−9.17%	1.9	485
	Baseline of Meta-ILF [37] ^(2023)^	−6.37%	-	-	-	1.4	441
	DILF [23] ^(2025)^ *	−8.07%	−18.06%	−20.45%	−10.87%	1.51	427
Adaptive	Meta-ILF [37] ^(2023)^	−6.77%	-	-	-	5.4	429
	PAILF-Net (Ours)	−7.89%	−18.25%	−19.15%	−10.59%	3.1	486

* Note that while DILF prioritizes structural adaptability for computational efficiency, our method focuses on parameter-level adaptation. These two strategies are complementary and can be integrated to further enhance overall coding performance. The superscript (year) for each approach indicates the publication year of the corresponding study.

**Table 3:** The detailed BD-rate (%) comparison between JVET-AC0194 and PAILF-Net under the AI configuration. Negative values denote bitrate savings, representing an improvement in coding efficiency over the baseline.

Class	Sequence	AC0194 [34]			PAILF-Net (Ours)			Δ=Ours−AC0194		
		Y	U	V	Y	U	V	Y	U	V
A1	*Tango2*	−7.21%	−21.05%	−22.51%	−7.90%	−24.36%	−26.05%	**−0.69%**	**−3.31%**	**−3.54%**
	*FoodMarket4*	−7.58%	−12.89%	−12.60%	−8.40%	−13.84%	−14.85%	**−0.82%**	**−0.95%**	**−2.25%**
	*Campfire*	−4.11%	−6.33%	−13.18%	−5.27%	−7.41%	−14.39%	**−1.16%**	**−1.08%**	**−1.21%**
A2	*CatRobot*	−7.70%	−18.52%	−19.82%	−8.57%	−22.32%	−22.82%	**−0.87%**	**−3.80%**	**−3.00%**
	*DaylightRoad2*	−5.93%	−20.74%	−12.73%	−6.88%	−25.86%	−23.79%	**−0.95%**	**−5.12%**	**−11.06%**
	*ParkRunning3*	−5.26%	−6.00%	−6.04%	−6.24%	−13.07%	−14.88%	**−0.98%**	**−7.07%**	**−8.84%**
B	*MarketPlace*	−5.85%	−15.18%	−17.48%	−6.56%	−16.82%	−17.64%	**−0.71%**	**−1.64%**	**−0.16%**
	*RitualDance*	−8.44%	−14.67%	−18.31%	−9.20%	−10.87%	−16.89%	**−0.76%**	3.80%	1.42%
	*Cactus*	−5.98%	−11.60%	−9.64%	−6.68%	−14.96%	−12.71%	**−0.70%**	**−3.36%**	**−3.07%**
	*BasketballDrive*	−6.81%	−18.41%	−17.98%	−7.72%	−20.19%	−21.99%	**−0.91%**	**−1.78%**	**−4.01%**
	*BQTerrace*	−4.73%	−14.69%	−15.43%	−5.41%	−26.56%	−19.17%	**−0.68%**	**−11.87%**	**−3.74%**
C	*BasketballDrill*	−10.22%	−20.82%	−22.00%	−11.44%	−24.29%	−24.71%	**−1.22%**	**−3.47%**	**−2.71%**
	*BQMall*	−8.42%	−16.50%	−20.14%	−9.05%	−19.19%	−22.16%	**−0.63%**	**−2.69%**	**−2.02%**
	*PartyScene*	−5.36%	−9.81%	−9.97%	−5.78%	−10.32%	−11.90%	**−0.42%**	**−0.51%**	**−1.93%**
	*RaceHorses*	−5.35%	−15.79%	−17.37%	−5.87%	−16.68%	−20.52%	**−0.52%**	**−0.89%**	**−3.15%**
E	*FourPeople*	−10.07%	−14.26%	−14.57%	−10.65%	−18.32%	−17.39%	**−0.58%**	**−4.06%**	**−2.82%**
	*Johnny*	−10.01%	−16.02%	−18.58%	−10.75%	−22.09%	−24.46%	**−0.74%**	**−6.07%**	**−5.88%**
	*KristenAndSara*	−8.97%	−15.57%	−14.52%	−9.66%	−21.39%	−18.39%	**−0.69%**	**−5.82%**	**−3.87%**
Class A1		−6.30%	−13.42%	−16.10%	−7.19%	−15.20%	−18.43%	**−0.89%**	**−1.78%**	**−2.33%**
Class A2		−6.30%	−15.09%	−12.86%	−7.23%	−20.42%	−20.50%	**−0.93%**	**−5.33%**	**−7.63%**
Class B		−6.36%	−14.91%	−15.77%	−7.11%	−17.88%	−17.68%	**−0.75%**	**−2.97%**	**−1.91%**
Class C		−7.34%	−15.73%	−17.37%	−8.04%	−17.62%	−19.82%	**−0.70%**	**−1.89%**	**−2.45%**
Class E		−9.68%	−15.28%	−15.89%	−10.35%	−20.60%	−20.08%	**−0.67%**	**−5.32%**	**−4.19%**
Average		−7.12%	−14.94%	−15.72%	−7.89%	−18.25%	−19.15%	**−0.77%**	**−3.31%**	**−3.43%**

**Table 4:** The detailed BD-rate (%) comparison between JVET-AC0194 and PAILF-Net under the random access (RA) configuration. Negative values denote bitrate savings, representing an improvement in coding efficiency over the baseline.

Class	Sequence	AC0194 [34]			PAILF-Net (Ours)			Δ=Ours−AC0194		
		Y	U	V	Y	U	V	Y	U	V
B	*MarketPlace*	−3.71%	−22.77%	−20.14%	−4.17%	−33.58%	−22.74%	**−0.46%**	**−10.81%**	**−2.60%**
	*RitualDance*	−5.53%	−19.06%	−19.13%	−6.14%	−22.34%	−19.13%	**−0.61%**	**−3.28%**	0.00%
	*Cactus*	−4.80%	−16.14%	−10.55%	−5.32%	−20.34%	−13.21%	**−0.52%**	**−4.20%**	**−2.67%**
	*BasketballDrive*	−5.07%	−12.91%	−13.45%	−5.61%	−15.80%	−17.91%	**−0.54%**	**−2.89%**	**−4.46%**
	*BQTerrace*	−6.09%	−16.03%	−20.55%	−6.75%	−27.13%	−26.09%	**−0.66%**	**−11.10%**	**−5.54%**
C	*BasketballDrill*	−4.74%	−17.91%	−16.36%	−5.43%	−20.90%	−17.77%	**−0.69%**	**−2.98%**	**−1.41%**
	*BQMall*	−4.85%	−17.93%	−16.24%	−5.27%	−20.53%	−17.98%	**−0.42%**	**−2.60%**	**−1.74%**
	*PartyScene*	−4.56%	−18.83%	−10.23%	−4.89%	−21.03%	−14.07%	**−0.33%**	**−2.20%**	**−3.84%**
	*RaceHorses*	−3.24%	−18.80%	−16.46%	−3.39%	−19.45%	−20.78%	**−0.15%**	**−0.65%**	**−4.32%**
Class B		−5.04%	−17.38%	−16.76%	−5.60%	−23.84%	−19.81%	**−0.56%**	**−6.46%**	**−3.05%**
Class C		−4.35%	−18.37%	−14.82%	−4.75%	−20.48%	−17.65%	**−0.43%**	**−2.98%**	**−2.87%**
Average		−4.73%	−17.82%	−15.90%	−5.22%	−22.35%	−18.85%	**−0.49%**	**−4.52%**	**−2.95%**

**Table 5:** The detailed BD-rate (%) comparison between JVET-AC0194 and PAILF-Net under the lowdelay B (LDB) configuration. Negative values denote bitrate savings, representing an improvement in coding efficiency over the baseline.

Class	Sequence	AC0194 [34]			PAILF-Net (Ours)			Δ=Ours−AC0194		
		Y	U	V	Y	U	V	Y	U	V
B	*MarketPlace*	−3.90%	−25.63%	−23.25%	−4.27%	−32.15%	−25.03%	**−0.37%**	**−6.52%**	**−1.77%**
	*RitualDance*	−5.90%	−17.70%	−16.99%	−6.45%	−20.17%	−16.03%	**−0.56%**	**−2.47%**	0.95%
	*Cactus*	−5.63%	−17.90%	−14.57%	−6.04%	−21.20%	−16.93%	**−0.41%**	**−3.30%**	**−2.36%**
	*BasketballDrive*	−5.51%	−13.95%	−15.25%	−6.31%	−16.60%	−19.02%	**−0.79%**	**−2.66%**	**−3.76%**
	*BQTerrace*	−6.20%	−21.00%	−25.47%	−6.84%	−32.51%	−30.14%	**−0.64%**	**−11.50%**	**−4.67%**
C	*BasketballDrill*	−5.81%	−18.06%	−16.97%	−6.71%	−21.18%	−18.49%	**−0.90%**	**−3.12%**	**−1.52%**
	*BQMall*	−5.55%	−19.85%	−17.66%	−6.14%	−23.36%	−20.12%	**−0.59%**	**−3.51%**	**−2.46%**
	*PartyScene*	−5.31%	−22.17%	−12.64%	−5.63%	−23.74%	−15.24%	**−0.32%**	**−1.57%**	**−2.60%**
	*RaceHorses*	−3.06%	−19.13%	−17.28%	−3.36%	−19.03%	−21.98%	**−0.30%**	0.10%	**−4.70%**
E	*FourPeople*	−8.65%	−15.97%	−16.29%	−9.06%	−21.23%	−20.57%	**−0.41%**	**−5.26%**	**−4.28%**
	*Johnny*	−9.30%	−18.86%	−23.36%	−9.95%	−27.50%	−29.44%	**−0.66%**	**−8.64%**	**−6.08%**
	*KristenAndSara*	−7.26%	−18.51%	−17.08%	−8.07%	−24.48%	−21.18%	**−0.82%**	**−5.97%**	**−4.10%**
Class B		−5.43%	−19.24%	−19.11%	−5.98%	−24.53%	−21.43%	**−0.55%**	**−5.29%**	**−2.32%**
Class C		−4.93%	−19.80%	−16.14%	−5.46%	−21.83%	−18.96%	**−0.53%**	**−2.03%**	**−2.82%**
Class E		−8.40%	−17.78%	−18.91%	−9.03%	−24.40%	−23.73%	**−0.63%**	**−6.62%**	**−4.82%**
Average		−6.01%	−19.06%	−18.07%	−6.57%	−23.60%	−21.18%	**−0.56%**	**−4.54%**	**−3.11%**

**Table 6:** Performance overview and interaction of the proposed key components in terms of BD-rate (%). Negative values denote bitrate savings, indicating an improvement in coding efficiency relative to the baseline.

Multi-Scale Adaptive Parameter	QP	Grad	Y	U	V	ΔY	ΔU	ΔV
✓	✓	✓	−7.89%	−18.25%	−19.15%	0%	0%	0%
✓	✓		−7.73%	−17.29%	−17.66%	0.16%	0.96%	1.49%
✓			−7.59%	−17.43%	−17.85%	0.3%	0.82%	1.3%
			−7.12%	−14.94%	−15.72%	0.77%	3.31%	3.43%

**Table 7:** Ablation study on the parameter-adaptive mechanism across individual modules. Negative values denote bitrate savings, indicating performance gains relative to the anchor.

Feature Extraction	Adaptive Backbone	Adaptive Reconstruction	Y	U	V	Average
✓	✓	✓	−7.89%	−18.25%	−19.15%	−10.59%
	✓		−7.68%	−17.03%	−18.08%	−10.15%
✓		✓	−7.35%	−16.24%	−17.05%	−9.67%

**Table 8:** Ablation study on the multi-scale mechanism and the number of parallel kernels *k* within MSPAConv and S-MSPAConv. Negative values denote bitrate savings, indicating performance gains relative to the baseline.

Class	PAILF-Net			w.o. Multi-Scale			PAILF-Net (k=8)		
	k=4; APP(1), AAP(2), AAP(4)			k=4; APP(1)			k=8; APP(1), AAP(2), AAP(4)		
	Y	U	V	Y	U	V	Y	U	V
A1	−7.19%	−15.20%	−18.43%	−6.82%	−14.98%	−17.05%	−7.41%	−16.03%	−19.42%
A2	−7.23%	−20.42%	−20.50%	−6.95%	−18.50%	−18.50%	−7.61%	−21.48%	−21.73%
B	−7.11%	−17.88%	−17.68%	−6.85%	−17.00%	−16.50%	−7.43%	−18.97%	−18.77%
C	−8.04%	−17.62%	−19.82%	−7.90%	−16.92%	−19.60%	−8.33%	−18.51%	−20.95%
E	−10.35%	−20.60%	−20.08%	−10.20%	−18.30%	−18.65%	−10.61%	−21.50%	−21.67%
Average	−7.89%	−18.25%	−19.15%	−7.73%	−17.14%	−18.06%	−8.19%	−19.22%	−20.34%
Complexity (kMACs/Pixel)	486			486			486		
#Parameters (M)	3.1			2.9			4.8		

**Table 9:** Ablation study on the incorporation of gradient grad and quantization parameters qp. Negative values denote bitrate savings, indicating performance gains relative to the baseline.

Class	PAILF-Net			w.o. grad			w.o. grad and qp		
	Y	U	V	Y	U	V	Y	U	V
A1	−7.19%	−15.20%	−18.43%	−7.14%	−14.80%	−17.29%	−6.75%	−15.04%	−16.90%
A2	−7.23%	−20.42%	−20.50%	−6.97%	−18.77%	−19.37%	−6.83%	−18.93%	−18.09%
B	−7.11%	−17.88%	−17.68%	−6.95%	−16.76%	−15.51%	−6.83%	−17.28%	−16.36%
C	−8.04%	−17.62%	−19.82%	−7.88%	−17.61%	−19.80%	−7.79%	−17.02%	−19.52%
E	−10.35%	−20.60%	−20.08%	−10.17%	−18.79%	−17.04%	−10.17%	−19.66%	−18.83%
Average	−7.89%	−18.25%	−19.15%	−7.73%	−17.29%	−17.66%	−7.59%	−17.43%	−17.85%

**Table 10:** Computational efficiency comparison in terms of encoding (Enc.) time and memory (Mem.) consumption on CPU under the AI configuration.

Class	Resolution	JVET-AC0194		PAILF-Net		Ratio	
		Enc. Time (sec.)	Mem. (MB)	Enc. Time (sec.)	Mem. (MB)	Enc.	Mem.
A1	3840 × 2160	2446.50	2299.93	2538.06	2469.22	1.04×	1.07×
A2	3840 × 2160	4241.23	2371.03	4335.47	2558.94	1.02×	1.08×
B	1920 × 1080	1128.50	902.98	1168.75	1101.23	1.04×	1.22×
C	832 × 480	398.92	517.02	420.96	714.70	1.06×	1.38×
Average	-	1301.00	1138.04	1351.07	1365.15	1.04×	1.20×

## Data Availability

The datasets generated during and/or analyzed during the current study are available from the corresponding author on reasonable request.
